# Supplementary material for: Amplified Drought Alters Leaf Litter Metabolome, Slows Down Litter Decomposition, and Modifies Home Field (Dis)Advantage in Three Mediterranean Forests
Source: Plants (Basel). 2022 Sep 30;11(19):2582. doi: 10.3390/plants11192582 (PMC9571106; doi:10.3390/plants11192582)
Supplement: Supplementary file 1 [file plants-11-02582-s001.zip › plants-1911410-supplementary.pdf]

Supporting informations

Figure S1. Rain exclusion devices in the three forests studied: a) *Quercus pubescens* forest at O_3HP, b) *Quercus ilex* at Puechabon and c) *Pinus halepensis* at Fontblanche (according [19]).

a)

b)

c)

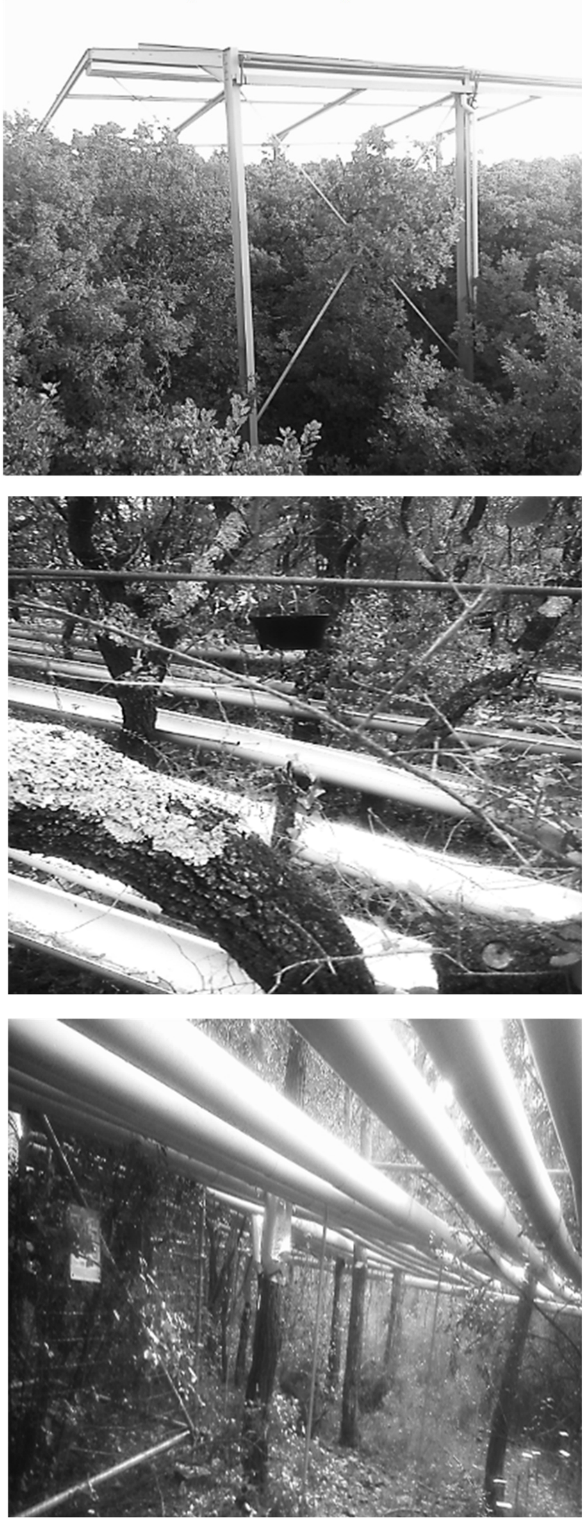

Figure S2. Ombrothermic diagrams at a) *Quercus pubescens* forest, b) *Quercus ilex* forest and c) *Pinus halepensis* forest study sites between December 2014 to December 2016. Dotted line and black triangle represent mean monthly temperature (°C) and lines represent mean monthly precipitation (mm) with black circle for ND plots and open circle for AD plots. ND: natural drought and AD: Amplified Drought (according [19])

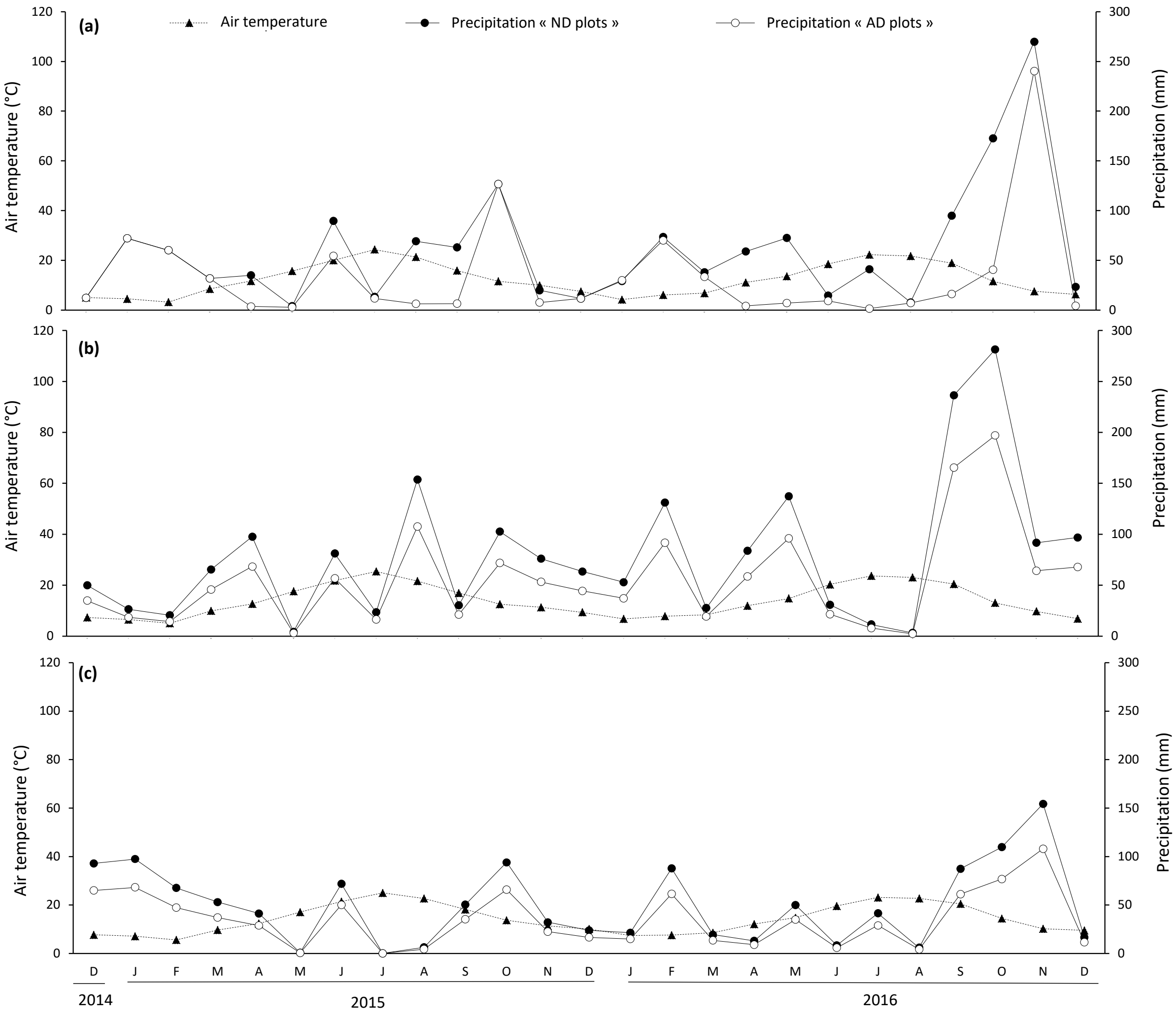

Figure S3. Location of the three forests studied: Quercus ilex forest (Puéchabon), Quercus pubescens forest (O3HP), and Pinus halepensis forest (Font blanche).

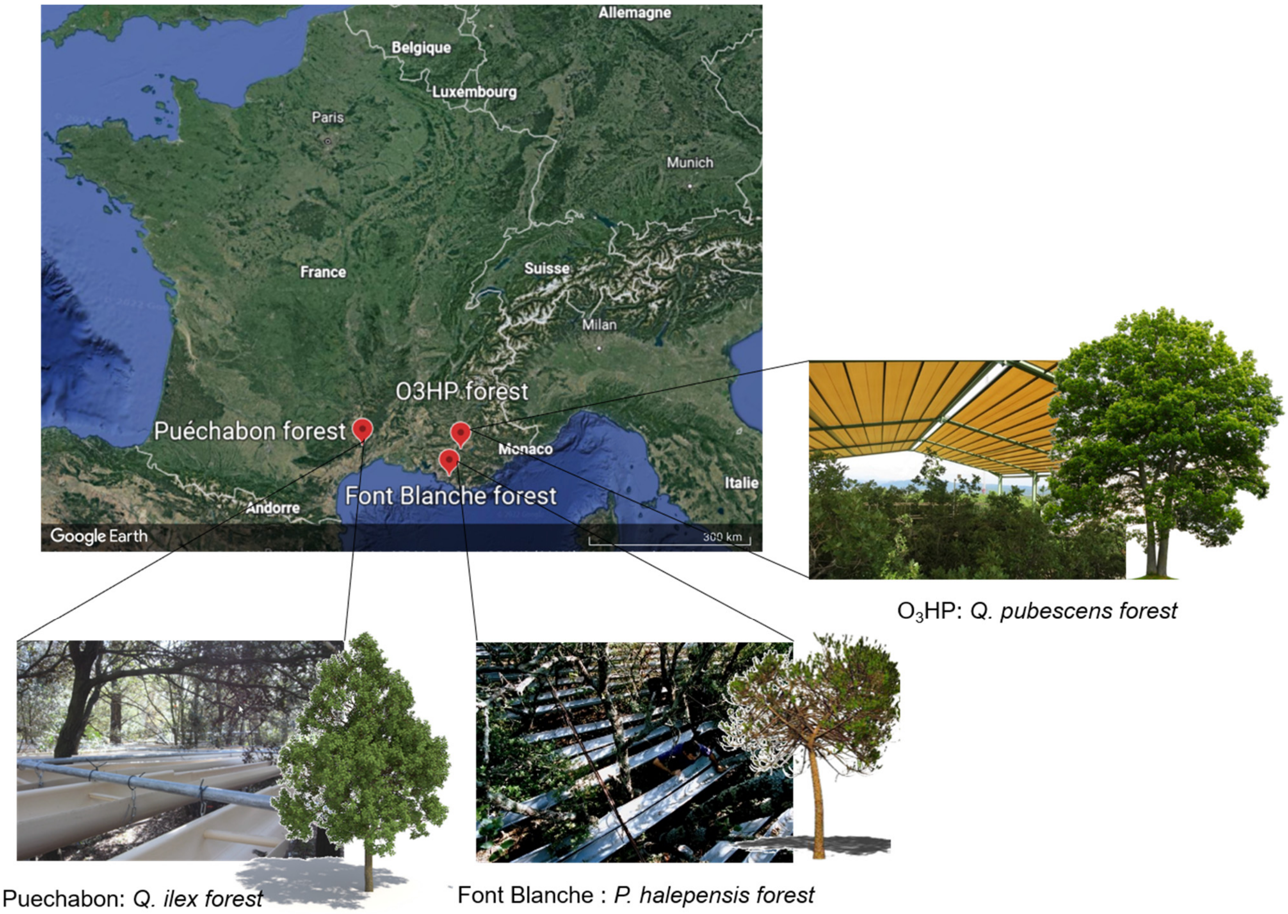

Table S1. Metabolites putatively identified from litter extracts of Q. ilex according to litter types (senescent leaves collected in Natural Drought (ND) and Amplified Drought (AD) plots). RT: Retention Time in minutes, VIP: Variable Important in Projection. mSigma is a constructor quality value of the formula determination. The closer the value is to zero, the more accurate the result.

Litter types	VIP	RT (min)	Measured m/z $[M+H]^+$	Measured m/z $[M-H]^-$	Measured molecular formula $[M-H]^-$	Error (PPM)	mSigma	MS/MS, m/z (ESI-)	Putative identification	Ref.
AD	M345T430	7.17	369.1903 $[M+Na]^+$	345.1553	$C_{16}H_{25}O_8$	1.1	9.9	326.9775<br>270.9846<br>215.2774<br>161.0462<br>101.0239	Unknown glycoside 1	
ND	M373T51	0.85	/	373.1349	$C_{13}H_{25}O_{12}$	0.4	21	NA	Unknown 1	
AD	M615T468	7.8	617.114	615.0987	$C_{28}H_{23}O_{16}$	0.9	9.1	463.0872<br>301.0348<br>300.0270	Quercetin-3-O-(6’’-O-galloyl)-b-D-glucopyranoside	[44]
AD	M629T496	8.26	631.129	629.1144	$C_{29}H_{25}O_{16}$	1.1	28	477.1025<br>315.0505<br>313.0567<br>169.0141	Isorhamnetin 3-(6"-galloylglucoside)	[44]
AD	M452T367	6.12	/	451.2183 $[M+HCOO]^-$	$C_{20}H_{35}0_{11}$			405.2131<br>167.1074	Unknown 2	
				405.2131	$C_{19}H_{33}O_9$	-0.3	6.7	167.1074		
AD	M340T388 (isotope)	6.47	341.086	339.0717	$C_{15}H_{15}O_9$	1.5	2.3	177.0192	Aesculin	[45]
ND	M387T49	0.81	/	387.1143 $[M+HCOO]^-$	$C_{13}H_{23}O_{13}$	0.5	11	341.1089<br>207.0511<br>179.0550<br>161.0459<br>119.0444<br>101.0235	Disaccharide	
				341.1086	$C_{12}H_{21}O_{11}$	0.9	13.8			

AD	M391T466	7.77	/	391.1037 [M+HCOO]$^-$	$C_{17}H_{27}O_{10}$	2.2	27.5	183.1025	Unknown glycoside 2	
				345.1557	$C_{16}H_{25}O_8$	-0.7	17.0			
AD	M330T66 (isotope)	1.09	/	331.0671	$C_{13}H_{15}O_{10}$	-0.5	21.6	169. 0138	galloyl-β-D-glucose	[44]
AD	M619T544	9.07	633.275 [M+Na]$^+$	619.2602	$C_{28}H_{43}O_{15}$	3.6	31.6	399.1301<br>339.1084<br>225.0770<br>195.0663	Unknown 3	
AD	M463T416	6.92	/	463.1822	$C_{20}H_{31}O_{12}$	4.2	25.2	180.9354<br>166.0657	Unknown (low intensity)	
AD	M399T407	6.78	355.101	399.0934	$C_{16}H_{17}O_9$	-3.9	NA	176.0104<br>191.0356	Chlorogenic acid	[46]<br>[47]<br>[48]
				353.0875	$C_{17}H_{19}O_1$	-3.3	23.0			
AD	M345T464	7.74	/	345.1554	$C_{16}H_{25}O_8$	0.9	14	183.1022<br>161.0450<br>165.0917<br>119.0350<br>113.0229<br>101.0240<br>85.0293	Unknown glycoside 3	
AD	M429T51	0.85	/	429.1252	$C_{15}H_{25}O_{14}$	1	22.3	NA	Unknown 4	
AD	M391T459	7.64	/	391.1607 [M+HCOO]$^-$	$C_{17}H_{27}O_{10}$	2.2	27.5	183.1025	Unknown glycoside 4	
				345.1554	$C_{16}H_{25}O_8$	0.8	12.3	183.1024 ($C_{10}H_{15}O_3$)		

Table S2. Metabolites putatively identified from litter extracts of Q. pubescens according to litter types (se-nescent leaves collected in Natural Drought (ND) and Amplified Drought (AD) plots). RT: Re-tention Time in minutes, VIP: Variable Important in Projection. mSigma is a constructor quality value of the formula determination. The closer the value is to zero, the more accurate the result.

Litter types	VIP	RT (min)	Measured m/z $[M+H]^+$	Measured m/z $[M-H]^-$	Measured molecular formula $[M-H]^-$	Error (PPM)	mSigma	MS/MS, m/z (ESI-)	Putative identification	Ref.
AD	M711T533	8.88	/	711.3964<br>$[M+HCOO]^-$	$C_{37}H_{59}O_{13}$	-0.5	25.8	503.3385	Arjungenin glycoside isomer 1	[49]
				665.3906	$C_{36}H_{57}O_{11}$	4.6	na			
AD	M243T473	7.87	/	243.1601	$C_{13}H_{23}O_4$	0	11.7	183.1391<br>117.0379	Unknown 1	
AD	M461T368	6.13	485.1279<br>$[M+Na]^+$	461.1302	$C_{19}H_{25}O_{13}$	-0.7	11.5	152.0113<br>108.0215	Unknown 2	
AD	M711T496	8.26	/	711.3965<br>$[M+HCOO]^-$	$C_{37}H_{59}0_{13}$	-0.9	14.9	/	Arjungenin glycoside isomer 2	[49]
				665.3906	$C_{36}H_{57}O_{11}$	-5.5	425.9			
AD	M427T396	6.59	/	427.1820<br>$[M+HCOO]^-$	$C_{17}H_{31}O_{12}$	-0.3	8.2	381.1782<br>299.0799<br>235.1193<br>187.0974<br>181.0524<br>144.0465	Unknown Glycoside	
				381.1774	$C_{16}H_{29}O_{10}$	1.9	77.7			
AD	M503T633	10.55	/	503.3382	$C_{30}H_{47}O_6$	-0.5	3.9	455.3178<br>304.9155<br>174.9552	Oleane triterpene	[50]
AD	M491T429	7.15	515.1897<br>$[M+Na]^+$	491.1408	$C_{20}H_{27}O_{14}$	-1.6	1.6	293.0887<br>151.0403	Unknown 3	
AD	M695T600	10.00	/	695.4018<br>$[M+HCOO]^-$	$C_{37}H_{59}O_{12}$	-1.4	6.9	487.3449	Arjunglucoside II or arjunetin	[49]<br>[51]
				649.3957	$C_{36}H_{57}O_{10}$	-2.7	NA			
AD	M431T383	6.38	/	431.1196	$C_{18}H_{23}O_{12}$	-0.1	11.3	137.0250<br>93.0346	Unknown 4	
ND	M387T49	0.81	/	387.1144	$C_{13}H_{23}O_{13}$	0.5	11	341.1086<br>179.0559<br>119.0349	Disaccharide	[48]
AD	M415T631	10.51	/	415.1320	$C_{22}H_{23}O_8$	0.8	27.6	400.1188<br>385.9936<br>341.1028<br>301.0361	Unknown 5	
ND	M702T383	6.38	/	701.5749	$C_{57}H_{46}O_{42}$	0.9	220.4	679.0745	Ellagitanin	[52]

	(isotope)			$[M-H]^{2-}$				617.0666<br>300.9991<br>249.0400<br>275.0191<br>231.0293<br>123.0083		
AD	M593T520	8.66	/	593.1591<br>$[M-H]^{2-}$	$C_{60}H_{50}O_{26}$	-1.6	24.7	285.0408	Proanthocyanidin (Cat-Cat-GalCat-GalCat)	[53]
AD	M343T637	10.61	/	343.0460	$C_{17}H_{11}O_8$	0.3	4.8	328.0216<br>312.9923<br>297.9722<br>286.9462<br>269.9814	Galloylquinic acid isomer	
AD	M289T405	6.74	291.086	289.0716	$C_{15}H_{14}O_6$	1.6	11.4	245.0823<br>221.0835<br>203.0720<br>151.0401<br>137.0246<br>123.0453<br>109.0293<br>97.0298	(epi)catechin	[54]
